# Stereotactic body radiation therapy for prostate cancer: systematic review and meta-analysis of prospective trials

**DOI:** 10.18632/oncotarget.27177

**Published:** 2019-09-24

**Authors:** Taylor R. Cushman, Vivek Verma, Rahul Khairnar, Joseph Levy, Charles B. Simone, Mark V. Mishra

**Affiliations:** ^1^ Department of Radiation Oncology, University of Texas M.D. Anderson Cancer Center, Houston, TX, USA; ^2^ Department of Radiation Oncology, Allegheny General Hospital, Pittsburgh, PA, USA; ^3^ Department of Health Policy and Management, Johns Hopkins Bloomberg School of Public Health, Baltimore, MD, USA; ^4^ Department of Radiation Oncology, University of Maryland School of Medicine, Baltimore, MD, USA

**Keywords:** prostate cancer, stereotactic body radiation therapy, stereotactic ablative radiation therapy, hypofractionation, toxicities

## Abstract

**Background:** Despite the increasing worldwide utilization of stereotactic body radiation therapy (SBRT) for prostate cancer, there are no known summative data regarding its safety and efficacy. To address this knowledge gap, we conducted a PRISMA-guided systematic review and meta-analysis of prospective prostate SBRT trials.

**Results:** Fourteen trials with a total of 2,038 patients were included. Median follow-up was 37 months (range 6-55 months). Most patients had cT1-T2a, Gleason ≤7 disease with median pre-treatment PSAs of 5–10; 1,042 (51%) were low-risk, 744 (37%) were intermediate-risk, 158 (8%) were high-risk, and the remainder were unreported. Doses ranged from 33.5–50.0 Gy, most typically in 5 fractions, with nearly all studies delivering nondaily fractionation with some type of daily image guidance. Outcomes were converted into counts at the end of one year. The pooled rate of FFBF was 98% [95% confidence interval, 97–98%]. The pooled rate of late grade ≥3 gastrointestinal and genitourinary toxicities were 1% [0–5%] and 2% [1–3%], respectively.

**Methods:** PubMed and Google Scholar were queried for prospective studies evaluating survival and/or toxicity outcomes in SBRT (≤5 fractions) for localized prostate cancer. Pooled rates of freedom from biochemical failure (FFBF) and late grades ≥3 gastrointestinal (GI) and genitourinary (GU) toxicities were assessed. Meta-analysis of proportions was logit transformed and pooled using generalized linear mixed models (both fixed and random effects) and subsequently back transformed to standard proportions.

**Conclusions:** Despite the lack of long-term follow-up and heterogeneity of the available evidence, prostate SBRT affords appropriate biochemical control with few high-grade toxicities. These data have implications for ongoing worldwide utilization of prostate SBRT as well as ongoing prospective investigations.

## INTRODUCTION

External beam radiation therapy (EBRT) for localized, non-metastatic prostate cancer (PC) has historically been delivered with conventionally fractionated doses of 1.8–2.0 Gy per fraction generally over the course of 40–44 treatments. However, given the inconvenience of this regimen for patients and concerns regarding the low α/β ratio of PC [1–4], prostate hypofractionation (most commonly 2.5–3.4 Gy per fraction) has come into better focus over the last decade, especially with the publication of randomized trials such as Radiation Therapy Oncology Group (RTOG) 0415, CHHIP, HYPRO, and PROFIT [5–8].

In addition to these encouraging phase III data, together with the expansion of technologies such as image guidance platforms, stereotactic body radiation therapy (SBRT) has become a highly active area of ongoing research for localized prostate cancer. SBRT also capitalizes on the low α/β ratio of PC and involves fractional doses even higher than hypofractionation. It is typically delivered in 5 or fewer fractions and allows for high target conformality in conjunction with high-quality image guidance. Dosimetric data even suggest that SBRT may be dosimetrically closer to high-dose-rate brachytherapy than non-stereotactic EBRT [9, 10].

Although the adoption of prostate hypofractionation is rapidly rising (and expected to expand even further in the future), there are few existing prospective experiences for SBRT, including no published phase III trials. In 2013, despite the limited data, the American Society for Radiation Oncology (ASTRO) listed SBRT as an alternative for low- and intermediate-risk PC [11]. The recently published guidelines from the American Urological Association, ASTRO, and the Society of Urologic Oncology also remain vague regarding the evidence-based utility of SBRT for PC [12].

However, in the absence of completed randomized trials, summative data are urgently needed in order to better evaluate the safety and efficacy of prostate SBRT. Hence, the goal of this work, the first of its kind to date, was to perform a systematic review and meta-analysis of prospective trials for prostate SBRT in PC.

## RESULTS

Supplementary Figure 1 displays a flow diagram of study selection. The initial search identified 612 studies, of which 24 were retrieved for full text review. Of those, 14 met the criteria of a prospective trial investigating survival and/or toxicity of SBRT in PC [15–32]. Publication year ranged from 2007 to 2017, and sample sizes ranged from 15 to 1,100 patients.

A summary of the study populations of the selected studies is displayed in Table 1. Of 2,038 total patients, 1,042 (51%) were low-risk, 744 (37%) intermediate-risk, and 158 (8%) high-risk; 91 (4%) did not have specific risk groups reported. Most patients had cT1-T2a, Gleason ≤7 disease with median pre-treatment prostate-specific antigen (PSA) values between 5 and 10.

**Table 1:** Summary of patient populations of the selected studies

Reference, Year	Sample Size	Risk Group Stratification (LR, IR, HR)	T Stage	Median pre-SBRT PSA	Gleason Grade	Baseline Symptoms or IPSS
Quon, 2018	152	20, 129, 0	1 (98), 2a (36), 2b (17)	7.2 (5.5–11.3), 8.2 (6.2–12.7)	6 (30) 7 (121)	7 (3–12), 4 (2–9)
Boyer, 2017	60	20, 40, 0	1c (47), 2a (11), 2b (2)	5.83	6 (24), 7 (36)	AUASS (4.5), EPIC (94.4)
Hannan, 2016	91	33, 58, 0	1c (63), 2a (20), 2b (8)	6.4	6 (43), 3+4 (33), 4+3 (15)	AUASS (5)
Rucinska, 2016	68	7, 61, 0	1c (6), 2a (15), 2b (19), 2c (28)	10	3 (2), 5 (21), 6 (14), 7 (29), 8 (2)	NR
Shikama, 2016	20	12, 8, 0	1c (16), 2a (2), 2b (1), 2c (1)	6.9	6 (14), 3+4 (3), 4+3 (3)	IPSS scores: 0–5 (12), 6–10 (4), 11–16 (4)
D’Agostino, 2016	90	53, 37, 0	NR	6.9	6 (58), 7 (32)	NR
Bauman, 2015	15	0, 0, 15	2 (11), 3 (5)	27.4	7 (9), 8-10 (6)	NR
Bernetich, 2014	142	61, 63, 18	1c (106), 2a (19), >2a (17)	5.7	5-6 (76), 7 (54), 8+ (12)	NR
Kim, 2014	91	—	1c (32), 2a (7), 2b (6)	5.6	6 (21), 3+4 (16), 4+3 (8)	NR
King, 2013	1100	641, 334, 125	NR	NR	NR	Median EPIC urinary score 89
Loblaw, 2013	84	84, 0, 0	1a (1), 1c (77), 2a (6)	5.3	6 for all	NR
Alongi, 2013	40	26, 14, 0	NR	6.25	6 (median)	IPSS 0-7
McBride, 2011	45	45, 0, 0	1c (33), 2a (12)	4.9	6 (45)	NR
Madsen, 2007	40	40, 0, 0	NR	5.0	<7 for all	Median AUA 13

Abbreviations: NR, not reported; LR, low-risk; IR, intermediate-risk; HR, high-risk; SBRT, stereotactic body radiation therapy; PSA, prostate-specific antigen; IPSS, International Prostate Symptom Score; AUASS; American Urologic Association Symptom Score; EPIC, Expanded Prostate Cancer Index.

Table 2 summarizes treatment-related parameters. The SBRT dose ranged from 33.5–50.0 Gy and was typically delivered in nondaily regimens. Image guidance was used in some capacity in all studies; although three did not report specific platforms, five studies utilized CyberKnife orthogonal radiography, five used cone-beam computed tomography (CT), and one utilized megavoltage CT. Eight studies allowed the use of androgen deprivation therapy (ADT) (nearly always ≤9 months). Only one study reported treatment of lymph nodes (which was hypofractionated) [26]. Management of rectal filling was incompletely reported by most studies.

**Table 2:** Summary of treatment parameters of the selected studies

Reference, Year	Sample Size	SBRT Dose, Fractionation, Timing	Image Guidance	ADT	LN Treatment	Rectal Management
Quon, 2018	152	40, q7d 40, q2d	CBCT	< 6 months	NR	NR
Boyer, 2017	60	37.5, 5, q2d	CBCT	None	None	Milk of Mg, fleet enema
Hannan, 2016	91	33.5, 5, q2d	NR	<9 months	None	NR
Rucinska, 2016	68	33.5, 5, q3d	MVCT	Yes, unspecified	None	NR
Shikama, 2016	20	35, 5, q2d	CyberKnife	≤ 8 months	NR	NR
D’Agostino, 2016	90	35, 5, q2d	CBCT	≤ 6 months	NR	NR
Bauman, 2015	15	40, 5, q1w	CBCT	≤ 12 months	25 Gy in 5 fx	NR
Bernetich, 2014	142	35/36.25/37, 5, q2d	CyberKnife	None	NR	NR
Kim, 2014	91	45/47.5/50, 5, q7d	CyberKnife	NR	NR	Milk of Mg + rectal balloon
King, 2013	1100	35–40, 5, qd	Cyberknife	≤ 3 months	None	NR
Loblaw, 2013	84	35, 5, q1w	NR	4 months	NR	NR
Alongi, 2013	40	35, 5, q2d	CBCT	Per NCCN guidelines	NR	SpaceOAR hydrogel
McBride, 2011	45	36.3–37.5, 5, qd	CyberKnife	None	NR	Yes, unspecified
Madsen, 2007	40	33.5, 5, qd	NR	NR	NR	NR

Abbreviations: NR, not reported; SBRT, stereotactic body radiation therapy; ADT, androgen deprivation therapy; LN, lymph node; CBCT, cone-beam computed tomography; Mg; magnesia; MVCT, megavoltage computed tomography; Gy, Gray; fx, fractions; NCCN, National Comprehensive Cancer Network.

**Figure 1:**
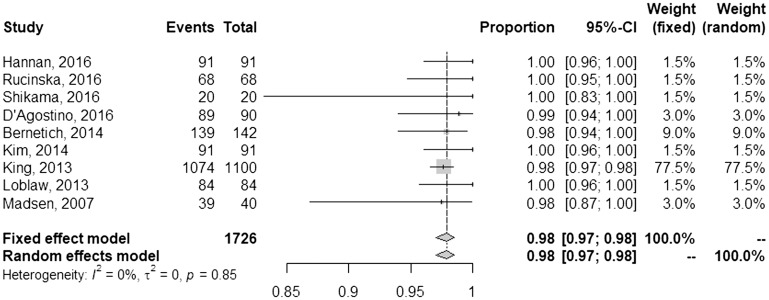
Pooled and individual rates of freedom from biochemical failure.

**Figure 2:**
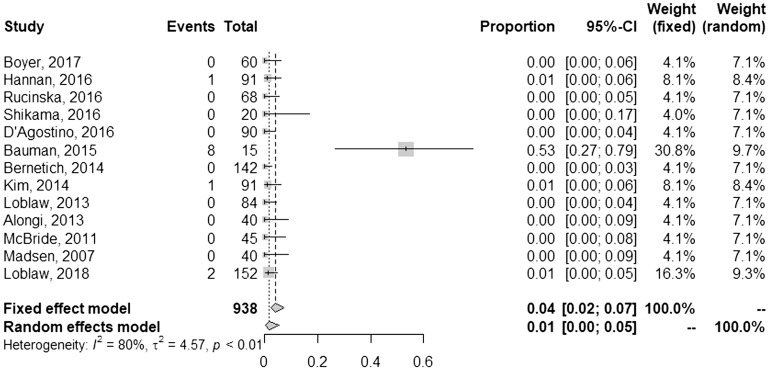
Pooled and individual rates of late grade ≥3 gastrointestinal toxicities.

**Figure 3:**
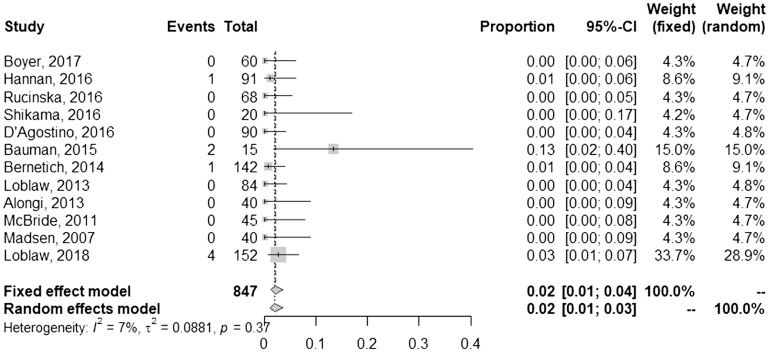
Pooled and individual rates of late grade ≥3 genitourinary toxicities.

A summary of outcomes is given in Table 3. Of the studies that documented a post-SBRT PSA nadir, most (75%) reported values of 0.5 or less. The mean and median follow-up of each study was 35 and 37 months, respectively. Using a random effects model, the pooled rate of FFBF at one year was 98% [95% confidence interval (CI), 97–98%] (Figure 1). Toxicities were reported using the Common Terminology Criteria for Adverse Events (CTCAE) for 8 studies, whereas 5 studies used RTOG methodology and one study did not report the method. Pooled late grade ≥3 gastrointestinal (GI) toxicity was 1% [95% CI, 0–5%] (Figure 2), and late grade ≥3 genitourinary (GU) toxicity was 2% [95% CI, 1–3%] (Figure 3). Nearly every study grouped both early and late adverse events; therefore, sub-analysis based on these factors could not be performed. Although quality of life parameters and measurements were highly heterogeneous, many studies reported a decline following SBRT that improved thereafter.

**Table 3:** Summary of outcomes of the selected studies

Reference, Year	Sample Size	Median follow-up (mo)	FFBF^*^ (% or median)	PSA Nadir	Toxicity Measurement Method	Late Grade ≥3 GI Toxicity (%)	Late Grade ≥3 GU Toxicity (%)	QOL Conclusions
Quon, 2018	152	47	NR	NR	RTOG	4.07	9.4	Prostate SBRT delivered QW improved acute bowel and urinary QOL compared to treatment EOD. Patients should be counselled regarding the significant short-term benefits of a longer overall treatment time.
Boyer, 2017	60	27.6	NR	NR	CTCAE	1.7	0	AUASS 11 during SBRT, and 5 at 5 mo EPIC 91.7 and 88.9 at 3 and 12 mo
Hannan, 2016	91	54	100% at 3y 98.6% at 5y	0.13	RTOG	6.8	5.5	No differences among dose levels for EPIC or AUASS
Rucinska, 2016	68	24	100%	0.03	RTOG	0	0	GHS/QoL was “good” 9 mo post-SBRT, significantly improved thereafter
Shikama, 2016	20	30	100%	0.73	CTCAE	2.5	0	NR
D’Agostino, 2016	90	28	97.8% at 27 mo	NR	CTCAE	0	0	NR
Bauman, 2015	15	6	NR	0.3	CTCAE	25	6.7%	NR
Bernetich, 2014	142	38	92.7% at 5 years for the entire cohort	0.16	RTOG	0	2	NR
Kim, 2014	91	42	99%	NR	CTCAE	5.5	NR	EPIC bowel scores lower than baseline 18 months post-SBRT
King, 2013	1100	36	93%	0.51	NR	NR	NR	Urinary and bowel QOL decline most notable within the first 3 mo, mostly recovered by 6 mo, stable thereafter, improvement over baseline starting at 3y
Loblaw, 2013	84	55	99.9%	NR	RTOG	1	1	No significant decline in long-term QOL
Alongi, 2013	40	11	NR	0.2	CTCAE	0	0	NR
McBride, 2011	45	45	NR	<1	CTCAE	0	2.5	Significant late decline in SHIM and EPIC sexual scores, small, late decline in EPIC bowel domain
Madsen, 2007	40	41	90%	NR	CTCAE	0	0	NR

Abbreviations: NR, not reported; FFBF, actuarial freedom from biochemical failure; PSA, prostate-specific antigen; GI, gastrointestinal; GU, genitourinary; QOL, quality of life; CTCAE, Common Toxicity Criteria for Adverse Events; AUASS; American Urologic Association Symptom Score; SBRT, stereotactic body radiation therapy; EPIC, Expanded Prostate Cancer Index; RTOG, Radiation Therapy Oncology Group; GHS, global health score; LR, low-risk; IR, intermediate-risk; HR, high-risk; SHIM, Sexual Health Inventory in Men.

^*^All studies but one utilized the Phoenix definition of biochemical failure.

## DISCUSSION

This systematic review and meta-analysis addresses a major knowledge gap and demonstrates that prostate SBRT produces appropriate biochemical control outcomes with few high-grade toxicities. However, these data should be interpreted with caution for several reasons, chiefly owing to the lack of long-term follow-up and heterogeneity of the available evidence. Nevertheless, this study has implications for ongoing utilization of prostate SBRT across the world as well as ongoing prospective trials.

There are several seminal conventional fractionation trials to which these data may be roughly compared, although available follow-up times remain a hindrance to interpretation. In a sentinel phase 3 trial of dose-escalation, Zietman and colleagues reported comparable toxicity outcomes and 10-year biochemical failure rates of 7.1% and 30.4% in low- and intermediate risk patients who received high-dose RT (79.2 Gy) [33]. The M. D. Anderson dose-escalation trial reported freedom from biochemical or clinical failure of 88% and 86% at 8 years in low- and intermediate risk patients in the dose-escalated (78 Gy) RT cohort, respectively [34]. Reported 10-year incidence of grade 3 GI and GU toxicities were 7% and 4%, respectively. Neither of these studies utilized contemporary image guidance or included ADT, and of note, the role of ADT in the setting of prostate SBRT for various risk groups is currently unknown. Nevertheless, the figures herein also compare well to more contemporarily published randomized trials of hypofractionated RT; however, limiting interpretation is the variability between trials regarding ADT, seminal vesicle target coverage, and most importantly, image guidance [5–8]. Nevertheless, when interpreted conservatively, data on prostate SBRT do not display an overt decrease in outcomes or increase toxicities compared with these historical conventional fractionation trials.

Consideration of strategies to reduce toxicities remains an integral factor in the radiotherapeutic treatment of PC, regardless of modality/technique, but especially important for SBRT. Foremost is the use of high-quality image guidance; to this extent, the use of megavoltage CT is likely inadequate for prostate SBRT, and kilovoltage fan-beam CT provides higher-quality imaging than kilovoltage cone-beam CT. Additionally, the vast majority of studies did not specifically report the degree of seminal vesicle target coverage or the methods for rectal management. As such, positive experiences with hydrogel rectal spacers in the conventionally fractionated setting may prove to be especially useful for prostate SBRT [35]. Additionally, nondaily fractionation regimens, seen in many of the studies included in analysis, may play a role in reduced toxicity [36]. One prospective trial used a unique fractionation delivery scheme of one fraction a week for eight weeks to 43.8–45.2 Gy [24] and reported no acute or late grade ³3 GI or GU toxicities, suggesting that an intermediate dose/fractionation scheme could be a satisfactory strategy for minimizing adverse events. Other considerations to reduce toxicities include ADT to reduce prostate volume prior to SBRT as well as more stringent selection of patients having undergone prior transurethral resection of the prostate.

Although this study is the largest investigation of SBRT for PC to date, the role of SBRT for PC will be better addressed with the eventual publication of multiple ongoing randomized trials. For instance, the international phase III Prostate Advances in Comparative Evidence (PACE) trial (NCT01584258) compares laparoscopic prostatectomy to SBRT and conventional RT or SBRT alone in patients with early-stage PC. Additionally, the HYPO-RT-PC trial (ISRCTN45905321) aims to compare the safety and efficacy of hypofractionated RT in intermediate-risk PC. Preliminary results display comparable late toxicity and favorable early toxicity of SBRT over standard fractionation [37]. However, it is important to note that this study utilizes seven fractions of 6.1 Gy each, and therefore would not meet the definition of SBRT (≤5 fractions) used in the current analysis.

The chief limitation of this work is the lack of long-term follow-up data in the analyzed trials; just two investigations herein reported median follow-up times approaching 5 years [15, 30]. Second, there are several sources of heterogeneity from study to study that are worth mentioning. For instance, there was a nearly even split in toxicity reporting based on ASTRO versus CTCAE criteria; research has highlighted the influence of reporting methodologies on differences in reported toxicity outcomes [38]. There were also various dose/fractionation schemes, baseline patient/disease characteristics, technical aspects of SBRT delivery, and image guidance capabilities. Prostate volume was also not discussed in nearly all studies, which may be an important determinant of toxicities [39]. Third, additional limitations of the analysis include the lack of individual patient data, as well as the fact that one study provided slightly over half the patients in this entire meta-analysis [20]. Moreover, as with any prospective study, the presence of enrollment bias may result in patients at higher risk of toxicities potentially not having been offered enrollment on protocol. Nevertheless, in light of these shortcomings, we encourage conservative interpretation of these data, while recognizing the necessity of long-term follow-up data.

## MATERIALS AND METHODS

### Systematic review

The systematic review was conducted using the Preferred Reporting Items for Systematic Review and Meta-Analyses [13] guidelines [13]. Eligibility criteria included prospective studies evaluating survival and/or toxicity outcomes with SBRT (defined as ≤5 fractions, the most common definition of SBRT used in the studies assessed) for non-metastatic PC.

The PubMed and Google Scholar search engines were queried for terms “stereotactic” or “SBRT” or “SABR” and “radiation” or “radiotherapy” and “prostate.” A broad keyword search was deliberately performed so as not to inadvertently exclude potentially relevant publications. Other data sources were publications known to the authors as well as those cited in relevant articles. In efforts to analyze only higher-quality evidence, only prospective trials were included; unpublished data were not included owing to the inability to verify methodology and validity. Care was taken to ensure that cohorts reported at multiple timepoints were not included more than once to avoid weighting bias; if there was overlap between publications from the same group, the most recent publication was utilized. Systematic searches were conducted by two independent authors. Date restrictions were not utilized and included all eligible articles published through January 1, 2018.

### Meta-analysis

For meta-analysis of proportions, data was logit transformed then pooled using an inverse variance methodology (both fixed and random effects), then back-transformed to standard proportions. Separate analyses were performed for the three main outcomes of the study: freedom from biochemical failure (FFBF), late grade ≥3 gastrointestinal (GI) toxicity, and late grade ≥3 genitourinary (GU) toxicity. To combine outcomes with varying endpoints, variables reported at median follow-up for each study were converted into counts at the end of year one, with the assumption of constant rates of occurrence over time. All analyses were conducted in R using the ‘metaprop’ function of the R package ‘metafor’, which calculates the overall proportion from studies reporting single proportions [14].

## SUPPLEMENTARY MATERIALS


