# Supplementary material for: Stereotactic body radiation therapy for prostate cancer: systematic review and meta-analysis of prospective trials
Source: Oncotarget. 2019 Sep 24;10(54):5660–8. doi: 10.18632/oncotarget.27177 (PMC6771455; doi:10.18632/oncotarget.27177)
Supplement: Supplementary file 1 [file oncotarget-10-5660-s001.pdf]

Stereotactic body radiation therapy for prostate cancer: systematic review and meta-analysis of prospective trials

SUPPLEMENTARY MATERIALS

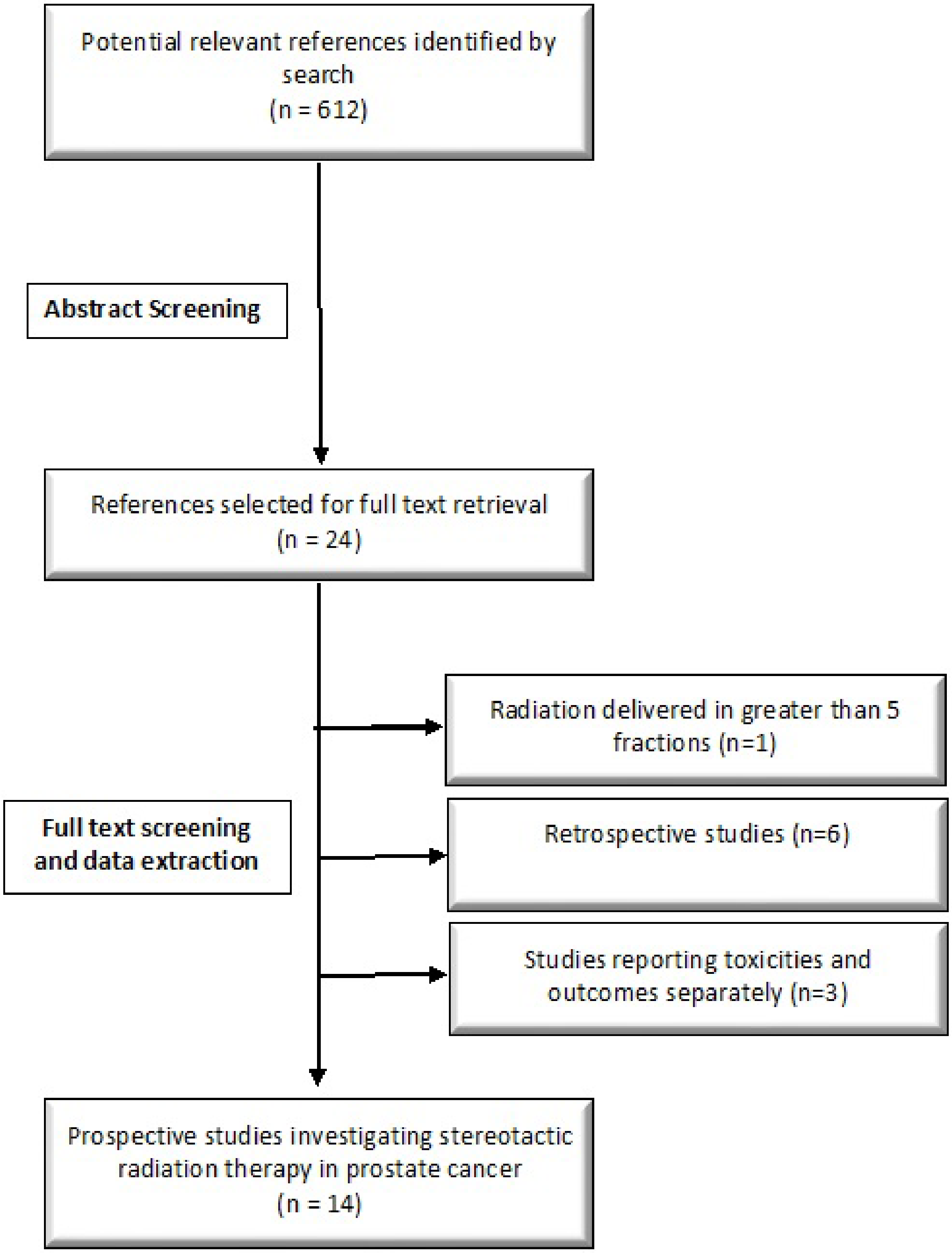

Supplementary Figure 1: Study selection diagram.
